# Rigby on the Constitutional Treatment of Female Diseases

**Published:** 1857-04

**Authors:** 


RIGBY *on the Constitutional Treatment of Female Diseases.*

This is a small-sized monograph, treating of those diseases peculiar to women. The subject is one of the highest importance, and the author's previous *System of Midwifery* a sufficient guarantee to the profession of his ability to render it interesting and instructive.

The first, second, and third chapters are devoted to the consideration of amenorrhœa, dysmenorrhœa, and menorrhagia. He enters as fully into the causes and treatment of these affections as the limits of such a work will admit. In the preface he says:—'I have devoted separate chapters to the consideration of these subjects [amenorrhœa, dysmenorrhœa, &c.] solely in deference to a long-established custom, which in former times was a necessity, when their nature and essential causes were imperfectly or erroneously understood, and which has been sanctioned by force of habit; but I feel assured that my readers will agree with me in the conviction that the time will come when these terms, as well as that of leucorrhœa will no longer designate distinct affections, but will be classed with such symptoms as pain, rigors, expectoration, &c.' The next chapter includes the consideration of 'Uterine and Vaginal Discharges,' which he divides into two classes:—*First*, Those connected with functional derangement; *Second*, Those connected with organic disease. The five following chapters he devotes to the study of inflammation, ulceration, and displacement of the uterus. His views on these subjects correspond with those of Sir C. M. Clark and Tyler Smith. The subsequent chapters comprise the consideration of simple aud malignant tumors of the uterus, and of the principal affections of the accessory organs of generation. Each of these subjects are treated with a brevity and interest such as to recommend the work to those whose limited opportunities will not admit of the perusal of a more complete treatise. The work is designed to be a practical one, and of utility to the practicing physician as a book of reference: as such, it claims due patronage.

